# Functional dynamics of bacterial species in the mouse gut microbiome revealed by metagenomic and metatranscriptomic analyses

**DOI:** 10.1371/journal.pone.0227886

**Published:** 2020-01-24

**Authors:** Youn Wook Chung, Ho-Jin Gwak, Sungmin Moon, Mina Rho, Ji-Hwan Ryu

**Affiliations:** 1 The Airway Mucus Institute, Yonsei University College of Medicine, Seoul, Korea; 2 Severance Biomedical Science Institute, Yonsei University College of Medicine, Seoul, Korea; 3 Department of Computer Science and Engineering, Hanyang University, Seoul, Korea; 4 Brain Korea 21 PLUS Project for Medical Science, Yonsei University College of Medicine, Seoul, Korea; 5 Department of Biomedical Informatics, Hanyang University, Seoul, Korea; University of North Texas, UNITED STATES

## Abstract

**Background:**

Microbial communities of the mouse gut have been extensively studied; however, their functional roles and regulation are yet to be elucidated. Metagenomic and metatranscriptomic analyses may allow us a comprehensive profiling of bacterial composition and functions of the complex gut microbiota. The present study aimed to investigate the active functions of the microbial communities in the murine cecum by analyzing both metagenomic and metatranscriptomic data on specific bacterial species within the microbial communities, in addition to the whole microbiome.

**Results:**

Bacterial composition of the healthy mouse gut microbiome was profiled using the following three different approaches: 16S rRNA-based profiling based on amplicon and shotgun sequencing data, and genome-based profiling based on shotgun sequencing data. Consistently, *Bacteroidetes*, *Firmicutes*, and *Deferribacteres* emerged as the major phyla. Based on NCBI taxonomy, *Muribaculaceae*, *Lachnospiraceae*, and *Deferribacteraceae* were the predominant families identified in each phylum. The genes for carbohydrate metabolism were upregulated in *Muribaculaceae*, while genes for cofactors and vitamin metabolism and amino acid metabolism were upregulated in *Deferribacteraceae*. The genes for translation were commonly enhanced in all three families. Notably, combined analysis of metagenomic and metatranscriptomic sequencing data revealed that the functions of translation and metabolism were largely upregulated in all three families in the mouse gut environment. The ratio of the genes in the metagenome and their expression in the metatranscriptome indicated higher expression of carbohydrate metabolism in *Muribaculum*, *Duncaniella*, and *Mucispirillum*.

**Conclusions:**

We demonstrated a fundamental methodology for linking genomic and transcriptomic datasets to examine functional activities of specific bacterial species in a complicated microbial environment. We investigated the normal flora of the mouse gut using three different approaches and identified *Muribaculaceae*, *Lachnospiraceae*, and *Deferribacteraceae* as the predominant families. The functional distribution of these families was reflected in the entire microbiome. By comparing the metagenomic and metatranscriptomic data, we found that the expression rates differed for different functional categories in the mouse gut environment. Application of these methods to track microbial transcription in individuals over time, or before and after administration of a specific stimulus will significantly facilitate future development of diagnostics and treatments.

## Introduction

With the emergence of high-throughput sequencing platforms, metagenomics has become a powerful approach for analyzing microbial communities [1, 2]. Traditional methods for profiling microbial composition rely primarily on targeted sequencing of 16S rRNA genes, which analyzes the relative abundance of the species in a microbial community [3, 4]. By contrast, shotgun sequencing is more effective in identifying the abundance of bacterial genes and their potential functions within a community, because it can decode the entire genetic material [5, 6]. Several large-scale surveys have been performed to find a possible association between the microbial composition and disease status. Nonetheless, analysis of the 16S rRNA genes or metagenome shotgun sequencing data is limited to the survey of bacterial composition in the microbiome of interest.

Shotgun sequencing of a metatranscriptome facilitates microbiome analysis with better resolution because different functional activities of individual genes in a species or in a microbial community can be explored under different conditions [7]. By mapping RNA sequencing reads to the known microbial genomes or a set of genes involved in a specific pathway, functional activity can be measured to find up- or down-regulated pathways in the microbial communities under various pathogenic conditions [5, 8–11].

Metatranscriptomic studies on the human gut microbiome revealed temporal changes of the microbial gene expression as well as actively transcribed genes or genes with suppressed functions [3, 12–15]. Some studies have documented the microbial dynamics of the human oral community during the dysbiosis of oral conditions [9, 16–18]. Recently, metatranscriptomic profiles of the human lung microbiome were obtained from patients with moderate and severe chronic obstructive pulmonary disease [15, 19]. Moreover, metatranscriptomic analysis of the gut microbiome during targeted exposure to xenobiotics [20, 21] and dietary changes [22] has been performed to discover significant alterations in the gene expression profiles, without significant changes in the overall community structure.

In the metagenomic analysis of the human gut microbiome, it is important to combine metatranscriptomic data. A recent study showed that several gene groups that are less abundant at the metagenomic level might be significantly active at the metatranscriptomic level, and vice versa [15]. This finding suggests that metagenomic analysis alone may overestimate or underestimate functional significance of the transcribed genes in the microbiome. For example, a metatranscriptomic analysis of 10 fecal samples from healthy volunteers has revealed that the phylogenetic composition is not evenly distributed among individuals [13]. In contrast, a functional analysis with the Cluster of Orthologous Groups (COG) database uncovered increased homogeneity in the distribution of the functional gene categories among the samples [13]. In another study on human oral microbiome, the relative abundance of bacterial genera obtained through metagenomic analysis was also different from that obtained by metatranscriptomic data [23]. Although recent studies have proved the association of community composition with the genomic potential of these microbiomes, how the genomic potential regulates transcriptional expression of the whole community is yet to be elucidated. To better understand the transcriptional effect on the microbiome, a combination of metagenomic and metatranscriptomic analyses is needed for specific species or strains within the complex gut microbiota.

Although mouse has been the primary choice as a model organism for functional studies, only a few studies have investigated the differential transcriptional ability of bacterial genes in the mouse gut microbiome under normal conditions. For example, metatranscriptomic analysis of the large intestine has been performed on a gnotobiotic mouse model system, which focused on a relatively simple community [22, 24].

In this study, we carried out a comprehensive analysis of metagenomic and metatranscriptomic data from the murine cecum. In order to find the expression patterns of the microbiome, pairs of the metagenome and metatranscriptome data were generated from eight mice. To estimate the bacterial composition in the gut microbiome, three different approaches comprising 16S rRNA profiling using amplicon sequencing data, 16S rRNA profiling using shotgun sequencing data, and genome-level profiling using shotgun sequencing data were employed for the metagenome data. In addition, the expression levels were quantified for the dominant species of the normal flora in the murine gut microbiome. Therefore, our results indicate a close relationship between the genomic potential and their expression in the mouse gut, and unveil important metatranscriptomic features in specific bacterial species in complex microbial communities.

## Results

### Bacterial composition of the mouse gut microbiome

We comprehensively investigated the bacterial composition of the gut microbiome from eight mice using three different profiling approaches and examined the gut microbiome of eight mice; 16S rRNA-based profiling with amplicon sequencing data, 16S rRNA-based profiling extracted from shotgun sequencing data, and genome-based profiling with shotgun sequencing data. Even though each approach has its own limitations, the overall composition revealed by the three approaches was consistent and complementary.

**Fig 1:**
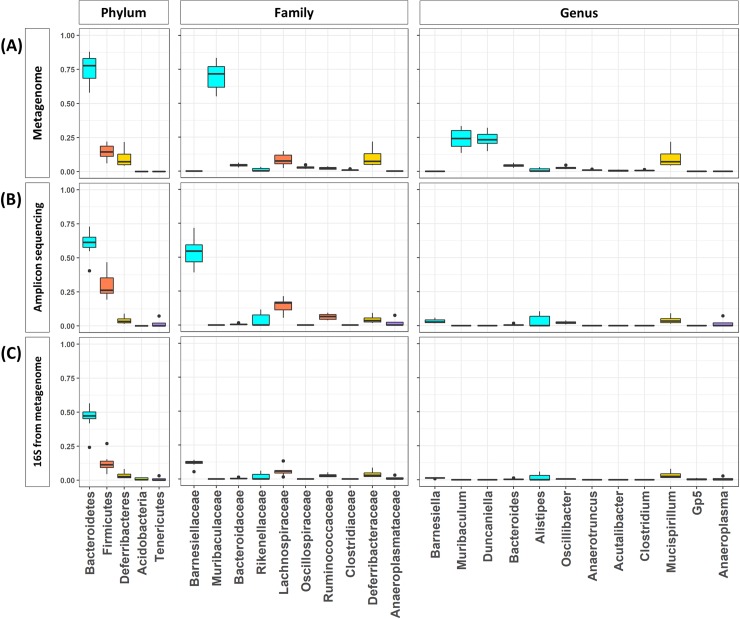
Bacterial composition of the eight mice gut microbiome estimated by three different approaches. Bacterial composition of the eight mice gut microbiome estimated by genome-based profiling using shotgun sequencing data (top), 16S rRNA-based profiling using amplicon sequencing data (middle), and 16S rRNA-based profiling using shotgun sequencing data (bottom). The color represents different phylum: cyan for Bacteroidetes, orange for Firmicutes, and yellow for Deferribacteres.

After we filtered out low-quality and host genomic reads, an average of 77.77% of the metagenome was retained (Table 1). Taxonomic assignment was performed at the phylum, family, and genus levels (Fig 1). Our analysis, using all three approaches, revealed that *Bacteroidetes* is the most abundant bacterium at the phylum level. Most *Bacteroidetes* strains were classified into the family *Muribaculaceae*, which constituted roughly 54.99% to 83.44% of the microbial community in the eight metagenome samples analyzed by genome-based profiling (Fig 1A). Notably, *Barnesiellaceae* was the most abundant family in 16S rRNA profiling based on amplicon sequencing and shotgun sequencing data (Fig 1B and 1C). This discrepancy is mainly due to the different versions of taxonomic classification that each method uses. The family *Muribaculaceae* is annotated in NCBI taxonomy and its genome could be identified, while this family is not included in the RDP classifier model. Therefore, the RDP classifier predicted this family as *Barnesiellaceae*, which is close to the family *Muribaculaceae*. In the whole metagenome analysis, we confirmed *Muribaculum* and *Duncaniella* (family *Muribaculaceae*), *Bacteroides* (family *Bacteroidaceae*), *and Alistipes* (family *Rikenellaceae*) as the predominant genera in the mouse gut microbiome (Fig 1A). The second most abundant phylum was *Firmicutes*, most of which was assigned to *Anaerotruncus* and *Oscillibacter* at the genus level (Fig 1A). The third most abundant phylum was revealed as *Deferribacteres*, which mostly consisted of *Mucispirillum* at the genus level (Fig 1A).

**Table 1:** Summary of metagenomic (MG) and metatranscriptomic (MT) data.

Samples	Raw reads	Low quality	Host genome	rRNA	Duplicated	Retained for analysis
MG1	37,250,272	1,173,619	3,432,398	152,067	1,390,026	31,102,162
MG2	37,922,399	1,158,675	1,785,568	165,400	1,463,627	33,349,129
MG3	42,289,523	2,874,989	1,267,331	178,398	2,001,801	35,967,004
MG4	34,517,538	1,168,846	4,671,768	137,557	1,107,499	27,431,868
MG5	33,129,291	860,283	4,393,498	141,944	1,277,661	26,455,905
MG6	68,973,711	1,209,460	11,239,904	291,189	2,932,048	53,301,110
MG7	64,390,225	1,665,760	5,799,291	300,742	2,630,213	53,994,219
MG8	38,200,675	293,686	10,501,277	146,684	1,266,447	25,992,581
MT1	51,518,586	39,385	846,427	105,464	9,465,557	41,061,753
MT2	54,356,835	39,075	1,631,150	144,028	10,226,812	42,315,770
MT3	52,252,893	137,428	883,052	120,324	9,685,672	41,426,417
MT4	58,256,982	40,854	315,347	95,836	12,617,154	45,187,791
MT5	50,986,731	50,246	340,820	76,258	9,771,730	40,747,677
MT6	55,380,658	137,955	15,735,360	293,084	3,358,470	35,855,789
MT7	51,412,952	47,921	10,655,569	256,501	6,858,291	33,594,670
MT8	49,895,941	50,853	1,933,768	127,292	10,423,191	37,360,837

Consistent with our findings, *Bacteroidetes* and *Firmicutes* have been reported as the most abundant phyla in the mouse gut microbiome in previous studies [24, 25]. Of note, *Deferribacteres* constituted a large proportion (4.26–21.78%) of our sample (Fig 1). In our analysis, higher proportions of *Deferribacteres* were observed in the whole metagenome analysis (average of 10.03%), compared to those in 16S rRNA profiling (average of 4.15% and 3.60% for amplicon sequencing and shotgun sequencing, respectively) (Fig 1). On the other hand, an extremely small proportion (~0.1%) was present in the previous study [25]. The top 20 core genera that were reported in the previous study [25] did not include any members of the *Mucispirillum* genus; this may be attributed to the use of an older version of the genome sequence database for metagenomic analysis in that study, since *Mucispirillum* has been recently sequenced, and therefore, it was not included in the earlier versions. To verify this notion, we re-analyzed the data from the report by Xiao et al. [25]. Twenty samples were randomly downloaded and their taxonomy profiles were estimated by metaphlan2. We confirmed that *M*. *schaedleri* was found in ten samples, constituting from 0.1% to 37% of the bacterial composition. Interestingly most of the *Firmicutes* were classified into *Clostridiaceae* and *Lachnospiraceae* in NOD mice, while *Lachnospiraceae* was observed to be the predominant family in our study (Fig 1). The effect of diet and housing on the bacterial composition has already been discussed in a previous study [24, 25].

### Distribution of functional contents encoded in the predominant genera of the mouse gut microbiome

**Fig 2:**
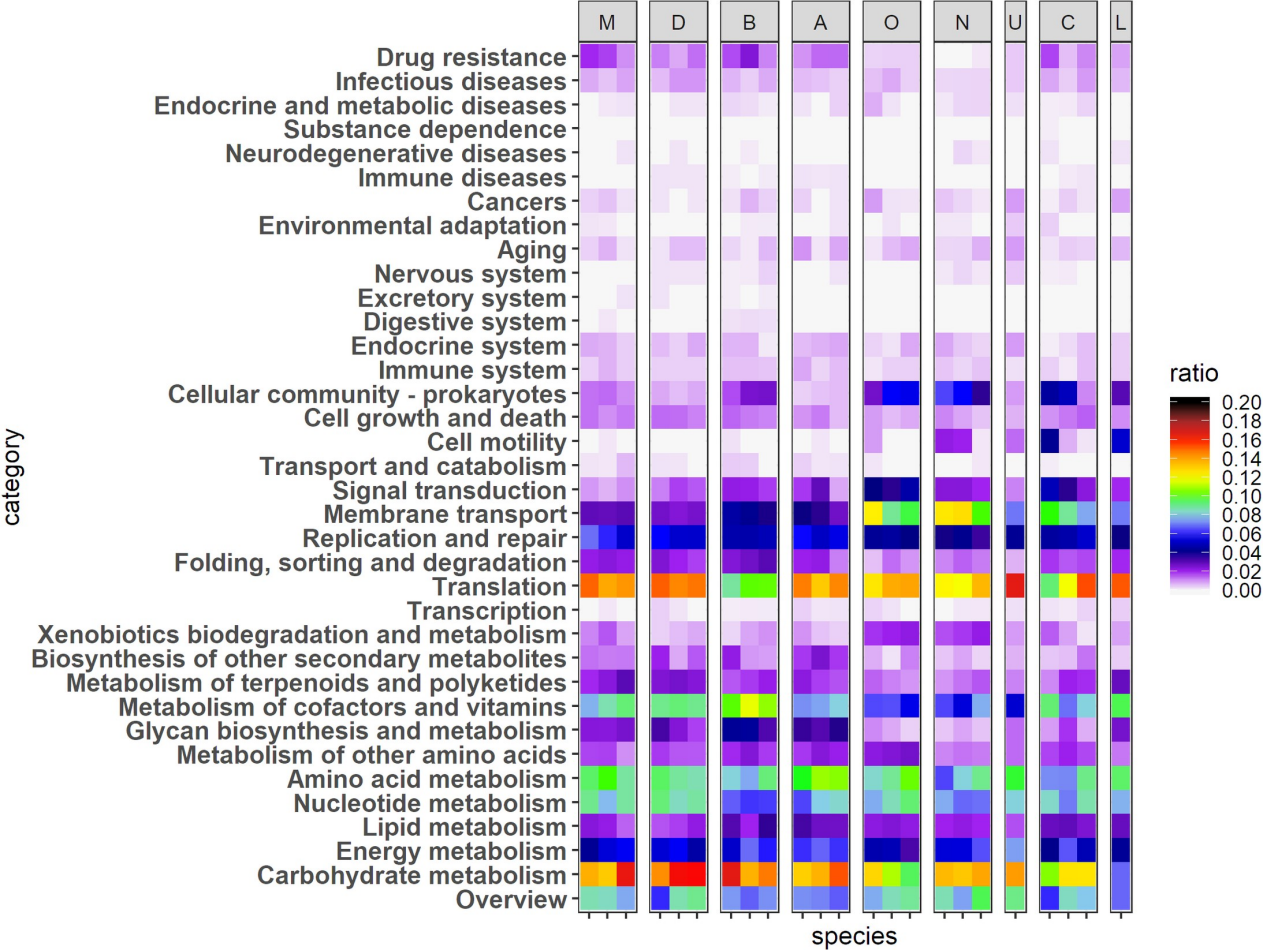
Abundance of functional contents encoded in the genomes of major nine genera in the mouse gut. Each column represents one species. Labels on the top represent each genus. M: Muribaculum, D: Duncaniella, B: Bacteroides, A: Alistipes, O: Oscillibacter, N: Anaerotruncus, U: Acutalibacter, C: Clostridium, and L: Mucispirillum. Specific species names are listed in Table 2. Each row represents a functional category in KEGG pathway. Each cell represents a ratio of genes in a specific genome, which is related to a given functional category.

The analysis of bacterial composition revealed that nine major genera constitute the mouse gut microbiome by genome-based profiling (Fig 1A). All the strains identified in each genus were analyzed to determine the functions encoded in the genome (S1 Fig). Each cell represents the ratio of a function encoded in a strain. Similar patterns of functional distribution were observed for most of the strains in each genus. Three strains were randomly selected manually from each genus (except two) for better comparison of the functions in the genome of nine genera (Fig 2, Table 2). *Acutalibacter* and *Mucispirillum* had only one strain each that were assembled at complete or scaffold level in NCBI repository (downloaded in 2019 May).

**Table 2:** Strains of the nine genera used for the analysis of functional distribution.

Species	Strain	Accession number	Assembly level	Size (Mb)
*Muribaculum intestinale*	YL27	GCA_002201515.1	Chromosome	3.31
*Muribaculum sp*.	H5	GCA_004803915.1	Chromosome	3.69
*Muribaculum sp*.	TLL-A4	GCA_004803695.1	Chromosome	3.43
*Duncaniella sp*.	B8	GCA_005304985.1	Complete	3.38
*Duncaniella sp*.	C9	GCA_004803935.1	Chromosome	3.42
*Duncaniella sp*.	TLL-A3	GCA_004766125.1	Scaffold	3.55
*Bacteroides eggerthii*	AM14-12	GCA_003472985.1	Scaffold	4.13
*Bacteroides ovatus*	OF05-12AC	GCA_003439865.1	Scaffold	6.72
*Bacteroides vulgatus*	AM35-11	GCA_003468485.1	Scaffold	5.17
*Alistipes indistinctus*	AF17-14	GCA_003460105.1	Scaffold	2.93
*Alistipes finegoldii*	DSM 17242	GCA_000265365.1	Complete	3.73
*Alistipes sp*.	Marseille-P5997	GCA_900604385.1	Complete	3.27
*Oscillibacter sp*.	1–3	GCA_000403435.2	Scaffold	4.47
*Oscillibacter sp*.	KLE 1745	GCA_000469445.2	Scaffold	3.59
*Oscillibacter sp*.	KLE 1728	GCA_000469425.1	Scaffold	3.59
*Anaerotruncus sp*.	AF02-27	GCA_003465835.1	Scaffold	3.74
*Anaerotruncus colihominis*	TF05-12AC	GCA_003435515.1	Scaffold	3.62
*Anaerotruncus colihominis*	2789STDY5834939	GCA_001404495.1	Scaffold	3.79
*Acutalibacter muris*	KB18	GCA_002201475.1	Chromosome	3.8
*Clostridium sp*.	AF27-2AA	GCA_003478505.1	Scaffold	3.58
*Clostridium sp*.	AF15-41	GCA_003604095.1	Scaffold	2.75
*Clostridium botulinum*	CDC_53174	GCA_001889345.1	Complete	3.87
*Mucispirillum schaedleri *	ASF457	GCA_000487995.1	Scaffold	2.33

Notably, *translation* and *carbohydrate metabolism* were the most abundant functions among 35 KEGG pathway categories in all nine genera (Fig 2). In *Bacteroides*, *carbohydrate metabolism* was the most abundant, while *translation* was less abundant, compared to the other eight genera. *Muribaculum*, *Duncaniella*, and *Alistipes* showed similar patterns of functional distribution, in which the amount of genomic content for *carbohydrate metabolism* was comparable with that for *translation* (14.46% vs 14.48% for *translation* and *carbohydrate metabolism*, respectively, in *Muribaculum*; 14.91% vs 15.57%, respectively, in *Duncaniella;* 14.16% vs 14.07%, respectively, in *Alistipes*) (Fig 2). The genomic content for *metabolism of cofactors and vitamins* was more enhanced in *Muribaculum*, *Duncaniella*, *Bacteroides*, and *Alistipes*, while in *Oscillibacter* and *Anaerotruncus* was this relatively small in number (Fig 2). Interestingly, the increase of genomic content for *membrane transport* was distinct in *Oscillibacter*, *Anaerotruncus*, and *Clostridium*.

### Differentially expressed genes of the predominant genera in the mouse gut

The expression of bacterial genes was quantified by all metagenomic and metatranscriptomic reads that were mapped against the reference genomes of the six most abundant species: *Muribaculum*, two species of *Duncaniella*, *B*. *caccae*, a speies of *Oscillibacter*, and *M*. *schaedleri*. Among the reference genomes in the NCBI repository, the closest strains were selected for each species. The abundance of each gene was measured by RPKM (Reads Per Kilobase transcript and per Million mapped reads) to determine the distribution of expression levels across the entire set of genes in each species. Considering the uneven abundance of genes in a species, which is due to the limitation of uneven sequencing, the expression levels of genes were investigated. Notably, the interquartile range of the gene abundance in the metagenomic data was narrow for most of the species, while the range of their expression levels was much wider in the metatranscriptomic data (S2 Fig).

**Fig 3:**
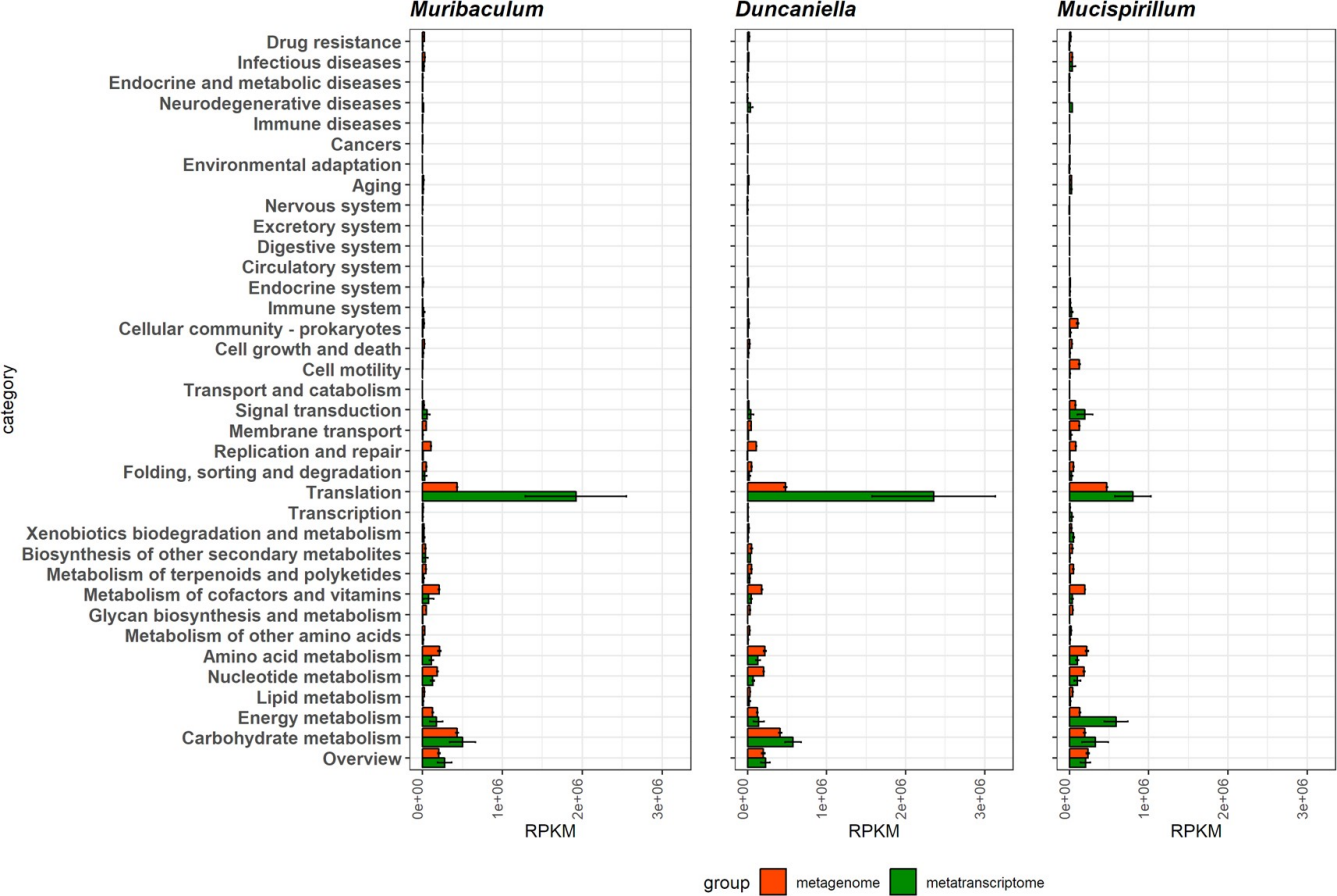
Abundance of functional contents measured from metagenomic and metatranscriptomic reads. Metagenomic and metatranscriptomic reads mapped to the top three genera (*Muribaculum*, *Duncaniella*, and *Mucispirillum*) were classified against the KEGG database. An abundance of functional contents encoded in the genome was measured from metagenomic reads (red) and expressed functional contents were measured from metatranscriptomic reads (green).

The quantification was further analyzed to characterize the functions of the normal flora in the mouse gut. For the three genera of *Muribaculum*, *Duncaniella*, and *Mucispirillum* that had enough reads covering more than 80% of the genome, the functional distribution was estimated based on the KEGG classification [26]. The reads of the metagenome and metatranscriptome that were mapped to the genera and the functions in the KEGG database, were quantified and normalized. Comparison of the function distributions of the metagenome with that of the metatranscriptome clearly revealed the differential expression of functions at the genomic and transcriptomic levels (Fig 3). As indicated by the functional analysis of the major genera (Fig 2), *translation* and *carbohydrate metabolism* were enhanced in the entire metagenome data (Fig 3).

*Translation* showed a distinctive increase in transcription, compared to the genomic contents in all three genera of *Muribaculum*, *Duncaniella*, and *Mucispirillum* (Fig 3). Though increased rate of metatranscriptome to metagenome of *carbohydrate metabolism* was lower than that of *translation*, expression level of *carbohydrate metabolism* was up-regulated in all three genera of *Muribaculum*, *Duncaniella*, and *Mucispirillum* (Fig 3). In contrast, expression level of *metabolism of cofactors and vitamins*, *amino acid metabolism*, and *nucleotide metabolism* was down-regulated in all three genera of *Muribaculum*, *Duncaniella*, and *Mucispirillum* (Fig 3). In case of *energy metabolism*, expression rate was not changed in *Muribaculum* and *Duncaniella*, but it was dramatically up-regulated in *Mucispirillum* (Fig 3). These results show that *translation* and *carbohydrate metabolism*, occupied significantly abundant gene contents in mouse gut microbiome, transcriptionally up-regulated in *Muribaculum*, *Duncaniella*, and *Mucispirillum*. In addition, increased or decreased pattern of transcriptional expression ratio is almost similar in *Muribaculum*, *Duncaniella*, and *Mucispirillum*.

### Functional dynamics of the metagenome and metatranscriptome

To investigate the functional distribution in the entire mouse gut microbiome with respect to the genomic contents and functional activity, both the metagenomic as well as the metatranscriptomic data were compared using KEGG database. Among seven categories at the highest level in KEGG pathways (*metabolism*, *genetic information processing*, *environmental information processing*, *cellular processes*, *organismal systems*, *human disease*, *and drug development*), *genetic information processing* function was the most dominantly expressed in transcriptomic data, which was due to the *translation* function (S3 Fig). In *genetic information processing* category, *translation*, and *replication and repair* functions were abundant (S3 Fig). In particular, *translation* was the significantly abundant function in the metatranscriptomic data, compared to the metagenomic data. However, *replication and repair* function was less abundant in the metatranscriptomic data. It ought to be noted that, this comparison was based on the relative abundance, indicating that the ratio of expressed transcripts among the entire transcripts in the metatranscriptomic data set was compared with the ratio of the genes among the entire genes in the metagenomic data set.

**Fig 4:**
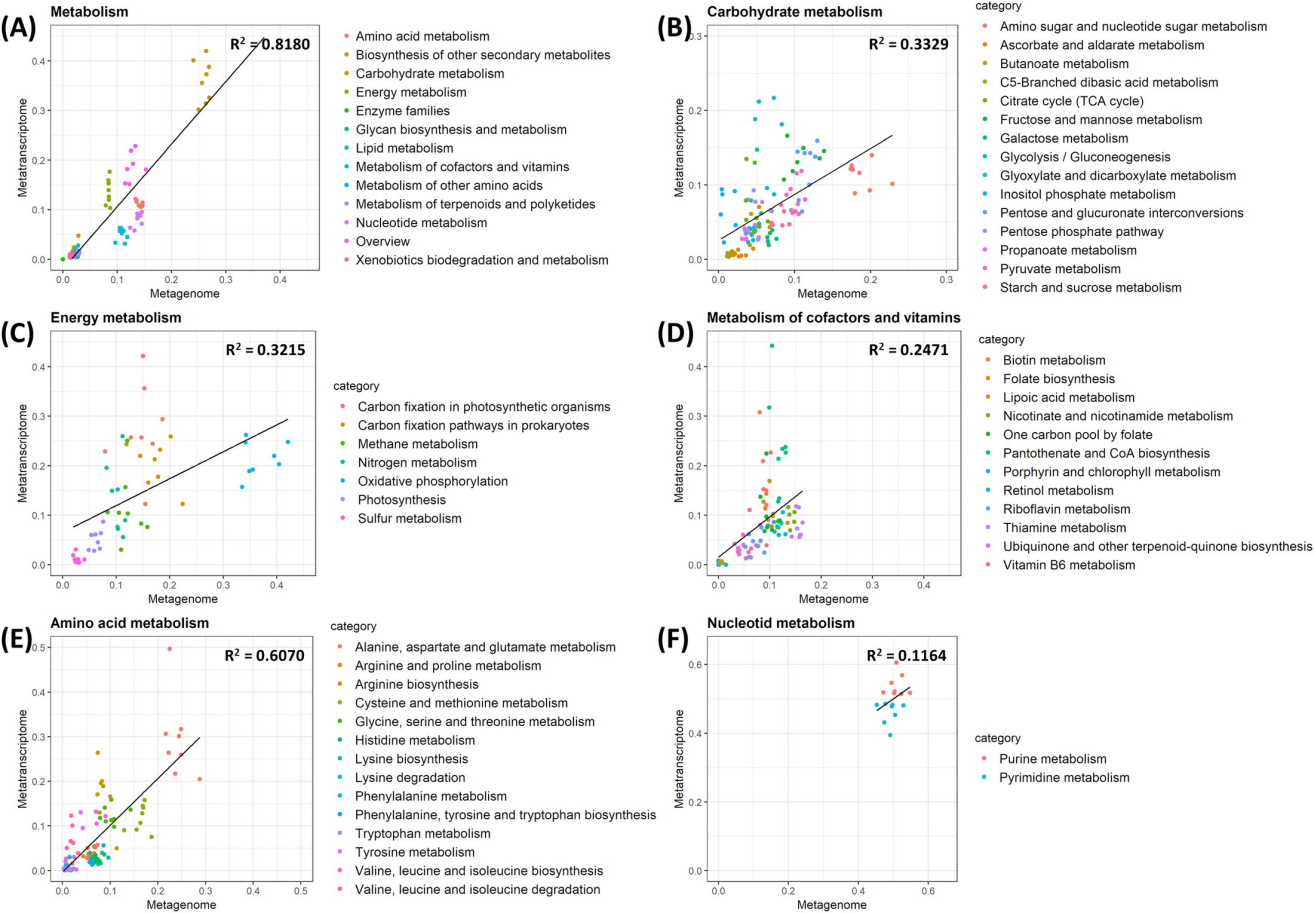
Relative abundance of genomic contents of metabolism and their expression activities in the metagenome and metatranscriptome. Relative abundance of each category (A) in metabolism, (B) in carbohydrate metabolism, (C) in energy metabolism, (D) in metabolism of cofactors and vitamins, (E) in amino acid metabolism, and (F) in nucleotide metabolism. The lines represent the relationship between the relative abundance of metagenome and metatranscriptome by linear regression.

Notably, *metabolism* functions constitute a large proportion of both the metagenomic and metatranscriptomic data. In particulr, *carbohydrate metabolism*, *amino acid metabolism*, *nucleotide metabolism*, *energy metabolism*, and *metabolism of cofacters and vitamins* were the abundant functions, which constituted more than 73.05% and 73.52% of metagenomic and metatranscriptomic reads, respectively that were assigned to the *metabolism* categories (Fig 4A). *Carbohydrate metabolism* and *energy metabolism* showed an evident increase in transcription, compared to the genomic contents. C*arbohydrate metabolism* constitutes 35.98% in transcriptomic contents, compared to 25.95% of *metabolism* in genomic contents (Fig 4A). *Energy metabolism* showed a similar pattern: 13.57% in transcriptomic contents vs 8.45% in genomic contents (Fig 4A). Among C*arbohydrate metabolism* pathways, *Glycolysis / Gluconeogenesis* and *Inositol phosphate metabolism* showed the higher expression rate compared to the genomic contents, while *amino sugar and nucleotide sugar metabolism* showed the lower expression rate (Fig 4B). Among the *energy metabolism* pathways, *Oxidative phosphorylation* showed the lower expression activity among seven functional categories that are defined in *energy metabolism* pathways (Fig 4C). On the contrary, *amino acid metabolism* and *nucleotide metabolism* showed a decrease in transcription (Fig 4A). *Amino acid metabolism* constitutes 13.99% of *metabolism* in genomic contents, and 10.95% in transcriptomic contents. *Nucleotide metabolism* constitutes 13.81% of *metabolism* in genomic contents, and 8.01% in transcriptomic contents. In *metabolism of cofacters and vitamins* pathway, *Pantothenate and CoA biosynthesis* and *Biotin metabolism* showed an increase in transcription (Fig 4D).

In order to profile the abundance of functional genes, genes were predicted from both the metagenomic and the metatranscriptomic data sets, and mapped against the KEGG protein database [26]. The number of non-redundant genes was tallied for each functional category (S4 Fig). The abundance of genes predicted from the assembled contigs revealed the genetic diversity rather than their expression levels in the metatranscriptomic data. This is because the reads sharing the origin are merged into a contig in the assembly process. The functions involved in *translation* and *carbohydrate metabolism* were highly encoded and expressed by the genomes. This result suggests that such functions are not only abundant but also highly expressed.

**Fig 5:**
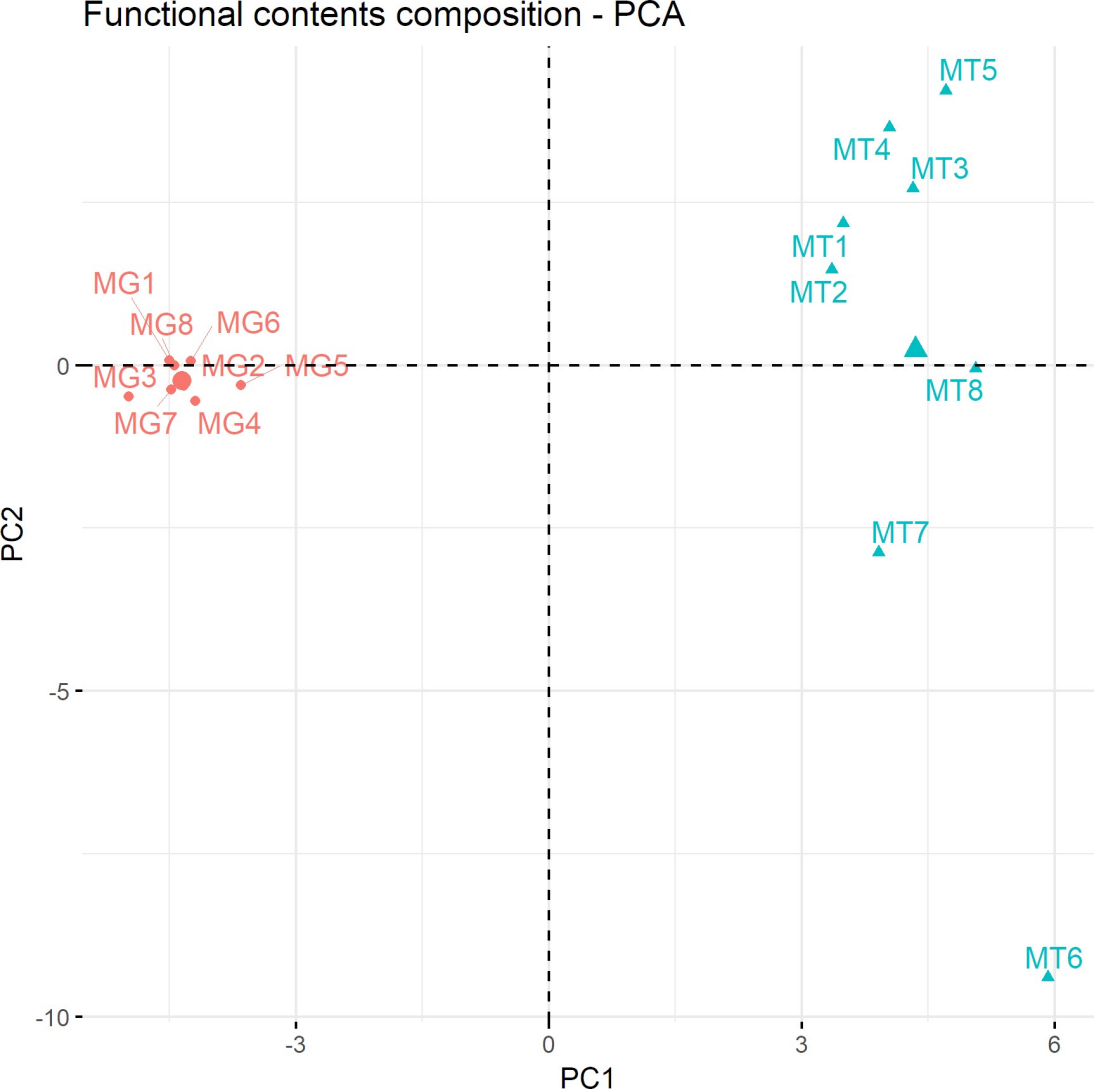
Principle component analysis of functional composition in metagenomic and metatranscriptomic data sets. Each sample was colored based on their source; metagenome sample is indicated by coral and metatranscriptome sample by mint color.

The functional diversity was compared among the metagenomic and metatranscriptomic data sets using principle component analysis (PCA) (Fig 5). The metagenomic data were closely clustered, while the metatranscriptomic data were more scattered. This result might imply that the functional activities of the microbiome are more dynamic in different mice even though the functional contents in the genome show similar patterns. In addition, we observed that the functional distribution in the transcriptomic data differed from that of the genomic data.

## Discussion

We comprehensively investigated bacterial composition of the mouse gut using amplicon sequencing and shotgun sequencing approaches. Our data revealed that *Bacteroidetes* was the most abundant bacterium at the phylum level and was classified into the following three major genera: *Muribaculum* and *Duncaniella* of the family *Muribaculaceae*, and *Bacteroides* of the family *Bacteroidaceae*. These findings were consistent with the murine gut microbiome composition determined previously by metagenomic shotgun sequencing [25]. The second major phylum was *Firmicutes*, which was mostly classified into *Anaerotruncus* and *Oscillibacter*. Xiao *et al*. also reported *Firmicutes* as the second most abundant phylum [25]. In our study, the third most abundant phylum was *Deferribacteres*, most of which was assigned to *Mucispirillum* at the genus level.

At the family level, *Barnesiellaceae* was the most abundant family in 16S rRNA-based profiling. This discrepancy is mainly due to the different versions of database for taxonomic classification that each method uses. *Muribaculaceae* genome is currently available in NCBI repository, which can be used as a reference genome. However, this family is not included in the current version of RDP classifier model. Therefore, the RDP classifier predicted this family as *Barnesiellaceae*, which is close enough to the family *Muribaculaceae*.

We also quantified the expression at the gene level by mapping all metagenomic and metatranscriptomic reads into reference genomes of the five most abundant species. The major genera play important roles in specific functions; for example, *Muribaculum* and *Duncaniella* (family *Bacteroidetes*) have more functions related to translation, whereas *Mucispirillum* (family *Deferribacteres*) has more functions for folding, sorting, degradation, and metabolism. These data indicate that the abundance of a gene family in the microbiota community appears to be a principal determinant of the abundance of its corresponding transcript.

To date, several reports have shown microbiota structure and microbiome function in the mouse gut under various environmental conditions by metatranscriptomic analysis [22, 24, 27–37]. In particular, metatranscriptomic analysis of the mouse gut microbiome during targeted exposure to lard and primary bile acid diet [29], a high-protein diet [31], and vitamin and mineral deficiencies [32] uncovered significant alterations in both bacterial community structure and their gene expression profiles. In addition to diet conditions, targeted infection with *Clostridium difficile* [30], specific exposure to organophosphate insecticides [34], and antibiotics [38] alter both the microbiome structure and its functional activity. In contrast, during a host gene deficiency, as in Perilipin-2 (Plin2)-deficient mice, high-fat diet causes the microbiota of Plin2-null mice to undergo significant shifts in transcript expression, despite no distinct change in overall community structure, as compared to wild-type mice [28]. Although the above reports point to the correlation between the whole-community composition and their genomic potential activity based on metatranscriptomic analysis, studies on how the genomic potential regulates the transcriptional expression in specific species or strains within a complex gut microbiota have not been conducted.

To the best of our knowledge, this is the first in-depth study to validate the functional activity of specific commensal microbes such as *Muribaculum*, *Duncaniella*, *Bacteroides*, and *Mucispirillum*, the most abundant genera within the mouse gut, via a combination of metagenomic and metatranscriptomic analyses. Different metatranscriptomic depth at various genes indicates that functional activities of the species are differently regulated in a gene-specific manner. Similarly, several genes dealing with bile acid metabolism in *B*. *vulgatus* are differentially expressed under a vitamin A deficiency condition [32]. During colorectal cancer (CRC) development, functional differences in the microbiome related to specific bacterial species, emerge from four pathways: lipopolysaccharide (LPS) production, polyamine synthesis, butyrate metabolism, and oxidative phosphorylation. Notably, an LPS production–related gene, specific for *M*. *schaedleri*, is dramatically upregulated in a CRC mouse model [33], supporting the differential expression of genes in *M*. *schaedleri*, also observed in our study.

The relative functional importance of genes in the gut of the living organism tends to be underestimated or overestimated by the metagenomics-only approach. Therefore, we examined transcriptionally up-regulated gene families by means of metagenomic and metatranscriptomic reads, mapped to those microbes. Based on functional classification via the KEGG database, the genes involved in translation, carbohydrate metabolism, and energy metabolism exhibited higher expression at the metatranscriptomic level, compared to that at the metagenomic level.

Consistent with the higher diversity and expression of ribosomal structure and biogenesis gene families in our metatranscriptomic data, microbial genes encoding ribosomal proteins are some of the most highly and variably expressed genes in either the human or mouse microbiome [15, 24]. The end products of ribosomal proteins are essential building blocks of the ribosome and must be constantly synthesized for survival of all microbes. Therefore, the high RNA expression level and their variability were caused largely by their high abundance at the DNA level. In addition, ribosomal genes are well conserved across species and strains. These represent a functional metatranscriptomic tool by which metagenomic stability (gene abundance and conservation) can be directly achieved in various host gut environments. We also observed that the carbohydrate metabolism gene families had much higher diversity and expression in the metatranscriptomic analysis, in line with earlier findings in both humans and mice [15, 24]. In general, metabolic routes including metabolism of carbohydrates such as starch and sucrose are unchanged or underexpressed, judging by the results of the metatranscriptomic analysis [13, 15]. In addition, many pathways related to the biosynthesis of small metabolites are expressed at relatively low levels [13, 15]. Given that these compounds have high bioavailability in the host gut owing to host diet, it seems reasonable that it would be more beneficial to transport them rather than to synthesize them in relation to energy efficiency. Notably, the less transcribed gene families in the metatranscriptomic analysis also have much lower abundance of metagenomes, except for gene families of replication, recombination, and repair. These data suggest that gene families relatively essential for microbial survival are likely to comprise higher abundance of genes exhibiting higher expression.

## Conclusions

In this study, we demonstrated a fundamental methodology for linking genomic and transcriptomic datasets to examine the functional activities of specific bacterial species in a complicated microbial environment. We investigated the normal flora of the mouse gut using three different approaches, and identified *Muribaculaceae*, *Lachnospiraceae*, and *Deferribacteraceae* as the predominant families. The overall bacterial composition was consistent, and the discrepancy was mostly due to the different taxonomic classifications used in the different databases. By comparing the metagenomic and metatranscriptomic data, we found that the expression rates differ for different functional categories in the mouse gut environment. In particular, *translation* and *carbohydrate metabolism* were the most abundant functions among 35 KEGG pathway categories in the most abundant nine genera. Application of these methods to track microbial transcription in individuals over time, or before and after administration of a specific stimulus, will significantly facilitate future development of diagnostics and pre/probiotic treatments.

## Materials and methods

### Preparation of samples

Eight-week-old wild-type C57BL/6 male mice were used in this study. Conventionally raised mice were housed in a specific pathogen-free (SPF) animal facilities under a 12 h light-dark cycle at 20±2°C and humidity range of 50±5% and maintained on normal chow diet (LabDiet 5053). Animals had access to diet and water *ad libitum*. Total eight mice were randomly grouped into three (3,2 and 3 mice). Each group of mice were anesthetized with zoletil-rompun mixture, a transverse abdominal incision was made, and the cecum was extracted. All animal experiments were performed according to the Guide for the Care and Use of Laboratory Animals and were approved by the Institutional Animal Care and Use Committee (IACUC) of Yonsei University Health System.

### Shotgun sequencing of the metagenome

Genomic DNA was isolated from murine cecal content using the QIAamp DNA Stool Mini Kit (Qiagen, Germany). The quantity and quality of DNA were assessed using PicoGreen dsDNA quantitation reagent (Invitrogen, Carlsbad, CA) and agarose gel electrophoresis, respectively. Each sequenced sample was prepared according to the Illumina protocols. Briefly, 100 ng of genomic DNA was fragmented into 350 bp inserts by means of a Covaris Focused-ultrasonicator (Covaris Inc., Woburn, MA). The fragmented DNA was blunt‐ended and phosphorylated. After the end repair process, the appropriate library size was selected using different proportions of the sample purification beads. A single ‘A’ base was ligated to the 3′ end of the fragmented DNA, followed by ligation of the Illumina adapters. The final ligated product was quantified by qPCR according to the qPCR Quantification Protocol Guide, and its quality was assessed using a 2200 TapeStation (Agilent Technologies, Palo Alto, CA). Sequencing was carried out on the HiSeq^™^ 4000 platform (Illumina, San Diego, CA).

### Shotgun sequencing of the metatranscriptome

Total RNA was extracted from mouse cecum using the Hybrid-RTM total RNA Purification Kit (GeneAll Biotechnology, Seoul, Korea). Concentration of total RNA was determined by Quant-IT RiboGreen (Invitrogen). To assess the integrity of total RNA, the samples were run on the TapeStation RNA ScreenTape (Agilent Technologies). Only high-quality RNA preparations, with RIN greater than 7.0, were used for RNA library construction. A library was prepared from each sample using 1 μg of total RNA with the Illumina TruSeq RNA Sample Prep kit (Illumina, Inc., San Diego, CA). In the first step, the poly‐A–containing mRNA of a mouse was removed by obtaining a supernatant by means of poly‐T oligo–conjugated magnetic beads. Thereafter, bacterial rRNA in the collected supernatant was depleted by Ribo-Zero bacteria. The remaining RNA was fragmented into small pieces using divalent cations at elevated temperature. The cleaved RNA fragments were transcribed into first-strand cDNA using SuperScript II Reverse Transcriptase (Invitrogen) and random primers. This step was followed by the second-strand cDNA synthesis, involving DNA Polymerase I and RNase H. These cDNA fragments were subjected to an end repair process, the addition of a single ‘A’ base, and ligation of the indexing adapters. The products were purified and enriched by PCR to create the final cDNA library. The libraries were quantified by qPCR according to the qPCR Quantification Protocol Guide (KAPA Library Quantification kits for Illumina Sequencing platforms). The quality of the libraries was assessed using the TapeStation D1000 ScreenTape (Agilent Technologies). The indexed libraries were sequenced by the Macrogen Inc. (Seoul, Korea) on the HiSeq 4000 platform (Illumina). All raw sequencing data described in this study are available at European Nucleotide Archive (ENA) with the accession number PRJEB33889.

### Processing of shotgun sequencing reads

Adapter sequences, low quality, and host reads were removed before the analysis. Adapter sequences were removed by SeqPurge, and low quality reads (whose Phred quality score was lower than 20) were discarded using Sickle; N-containing reads were discarded by in-house scripts. Therefore, an average of 3.01% and 0.13% of reads were discarded from the metagenomic and metatranscriptomic samples, respectively (Table 1). To remove the host reads, the retained reads were mapped against the USCS mouse reference genome (mm10) using Bowtie [39]. Reads that were mapped with two or fewer mismatches were discarded. Consequently, an average of 12.04% and 7.56% of reads were discarded from metagenomic and metatranscriptomic samples, respectively. In addition, the metatranscriptome reads were mapped against mouse mRNA sequences to remove the reads. An average of 0.02% and 0.01% of reads were discarded. Finally, ribosomal RNA reads were filtered out by SortMeRNA and duplicated reads were removed by FastUniq. An average of 0.42% and 0.29% of reads were filtered as rRNA reads and 3.88% and 17.11% of reads were removed as duplicated reads.

Contigs were assembled from each metagenomic and metatranscriptomic sequence using megahit [40] with default parameters. In each contig, genes were predicted using FragGeneScan [41] with the Illumina_5 error model.

For reads classified as rRNA, 16S rRNA reads were obtained by SortMeRNA using only the 16S rRNA database. The 16S rRNA reads that were retained, were used for the analysis of bacterial composition.

To obtain targeted variable region, amplicon sequencing reads were assembled by FLASH, allowing 20 to 300 bp overlap. Incorrectly assembled fragments, whose lengths were shorter than 380 bp, were filtered out by Sickle.

### Examination of bacterial composition

Operational Taxonomic Units (OTUs) were constructed for 16S rRNA-based profiling by cd-hit with 99% sequence similarity threshold. Their representative sequences were classified by RDP (Ribosomal Database Project) naïve Bayesian classifier [42], which classified all inputs from the phylum to the genus level and reported their scores. Only those classification results whose confidence scores were over 0.8 by default were considered in order to avoid misclassification. OTUs were classified at the lowest level and bacterial composition was estimated based on the classification results.

To analyze the bacterial composition using the metagenomic sequences, metagenomic reads were searched using the NCBI reference genomes. All reference sequences (complete- and scaffold-level assembly) were downloaded from the NCBI RefSeq database. For initial screening, metagenomic reads were searched against all proteins using DIAMOND [43] (cut off of e-value 1.0e^-10^, percent identity 70%, and query coverage 70%). The references to which reads were mapped were retained for more detailed analysis. Metagenomic reads were searched against the selected reference genomes using Bowtie [39]. Best hits whose percent identities were higher than 85% were retained. Aligned reads were classified based on the NCBI taxonomy and bacterial composition was estimated through tallying up the mapped reads.

To estimate bacterial composition in the data from the report by *Xiao et al*., 20 samples (ERR675499, ERR675503, ERR675507, ERR675513, ERR675525, ERR675553, ERR675554, ERR675575, ERR675584, ERR675600, ERR675607, ERR675610, ERR675623, ERR675638, ERR675652, ERR675655, ERR675667, ERR675672, ERR675679, ERR675687) were downloaded. MetaPhlan2 was performed with the default parameter setting except *ignore* flags (—ignore-archaea,—ignore-eukaryotes,—ignore-viruses) to determine whehter *Mucispirillum* exists or not.

### Examination of functional contents

To examine the functional contents, processed reads and proteins were searched against KEGG protein database using DIAMOND [43] (percent identity and query coverage cutoffs were set as 70% and 70%, respectively, for read search, and 50% and 50%, respectively, for protein search; e-value cutoff was 1.0e^-10^ for both searches). Database proteins that have no KEGG Ortholog (KO) number or have KO number without functional classification were discarded. For reads, RPKM values were calculated for all proteins and these values were tallied up to functional categories. RPKM is defined as below.

$$RPKM=x\times 10^{9}/\left(y\times z\right)$$,

where x is the number of reads mapped to the gene, y is the length of the gene sequence, and z is a total number of mapped reads in the sample.

For proteins, the number of mapped reads was counted for all proteins; the counts were tallied up to functional categories, and normalized by total number of mapped reads. Most proteins in the KEGG database have a single KO number, with only a few proteins having multiple KO numbers. In such cases, one KO was randomly chosen.

For statistical analysis of expression level, t-test was performed by using python statistics library of scipy.stats. For regression analysis, functions lm was used from standard R package.

## Supporting information

S1 Fig Abundance of functional contents encoded in each genus. Each column represents one species. Labels on the top represent each genus. *Muribaculum* (A), *Duncaniella* (B), *Bacteroides* (C), *Alistipes* (D), *Oscillibacter* (E), *Anaerotruncus* (F), and *Clostridium* (G). Each row represents a functional category in KEGG pathway.
(TIF)

S2 Fig Distribution of RPKMs for genes in six predominant species. (TIF)

S3 Fig Read-based abundance of genomic contents and functional activities of each mouse gut microbiome. Abundance was measured from the entire metagenomic and metatranscriptomic reads sequenced from each mouse gut.
(TIF)

S4 Fig Gene-based diversity of genomic contents and functional activities of each mouse gut microbiome. Abundance was measured from the number of non-redundant genes predicted from each mouse gut.
(TIF)

## Abbreviations

NOD: non obese diabetic
RPKM: reads per kilobase of transcript per million mapped reads
KEGG: Kyoto Encyclopedia of Genes and Genomes
KO: KEGG ortholog
Plin2: perilipin-2
CRC: colorectal cancer
LPS: lipopolysaccharide
SPF: Specific pathogen-free
RPM: Reads per million
