# Supplementary figures and images for: Functional dynamics of bacterial species in the mouse gut microbiome revealed by metagenomic and metatranscriptomic analyses
Source: PLoS One. 2020 Jan 24;15(1):e0227886. doi: 10.1371/journal.pone.0227886 (PMC6980644; doi:10.1371/journal.pone.0227886)

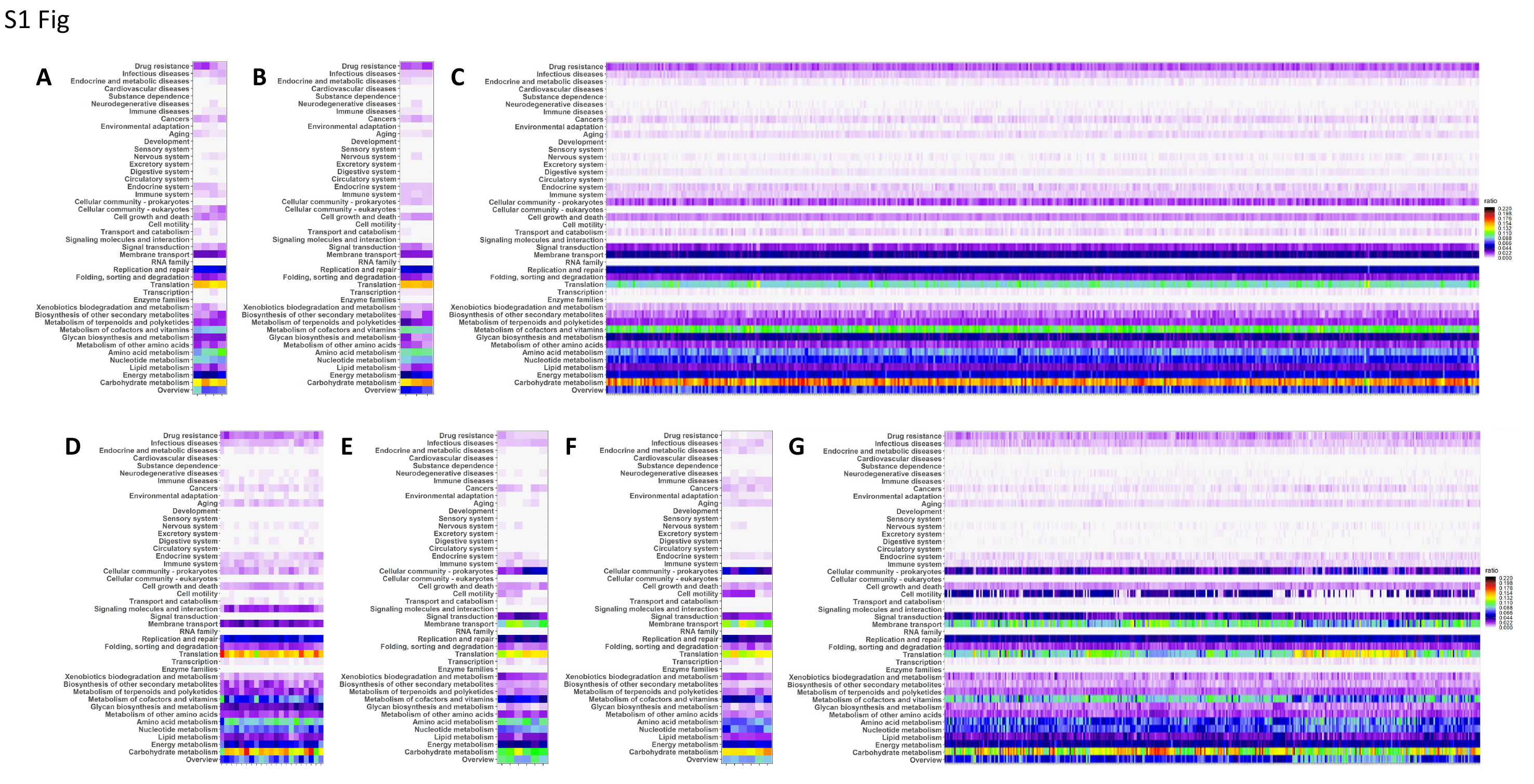

Supplement: S1 Fig — Each column represents one species. Labels on the top represent each genus. Muribaculum (A), Duncaniella (B), Bacteroides (C), Alistipes (D), Oscillibacter (E), Anaerotruncus (F), and Clostridium (G). Each row represents a functional category in KEGG pathway. (TIF) [file pone.0227886.s001.tif]

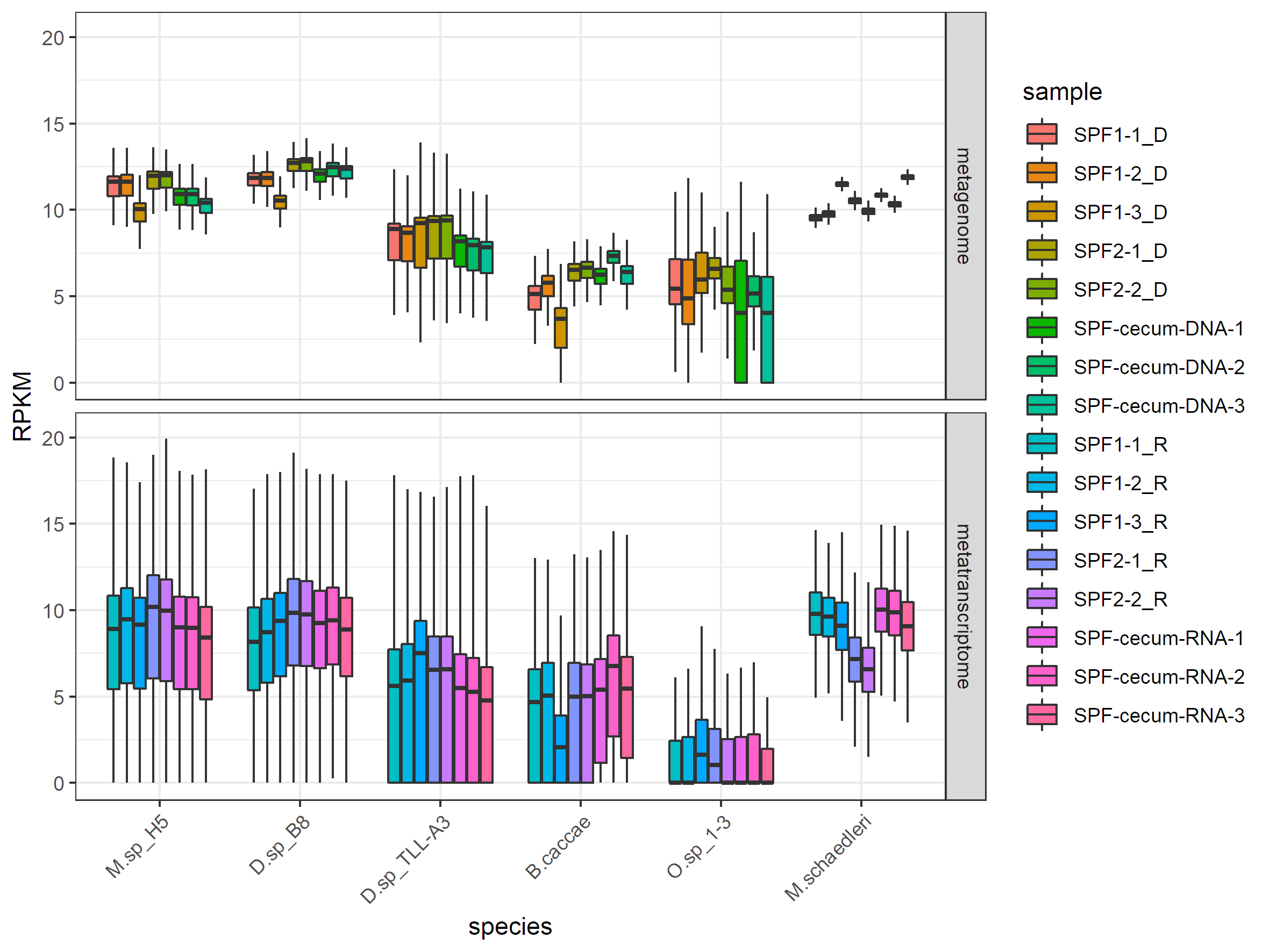

Supplement: S2 Fig — (TIF) [file pone.0227886.s002.tif]
